# High-level expression and purification of soluble recombinant FGF21 protein by SUMO fusion in *Escherichia coli*

**DOI:** 10.1186/1472-6750-10-14

**Published:** 2010-02-17

**Authors:** Huiyan Wang, Yechen Xiao, Lianjun Fu, Hongxin Zhao, Yaofang Zhang, Xiaoshan Wan, Yuxia Qin, Yadong Huang, Hongchang Gao, Xiaokun Li

**Affiliations:** 1 Engineering Research Center of Bioreactor and Pharmaceutical Development, Ministry of Education, Jilin Agricultural University, 130118, China; 2 School of Pharmacy, Wenzhou Medical College, Wenzhou, Zhejiang, 325000, China; 3 Jilin Agricultural Science and Technology College, 132101, China; 4 Biopharmaceutical research and Development Center, Institute of Life and Health Engineering, Jinan University, Guangzhou, 510642, China

## Abstract

**Background:**

Fibroblast growth factor 21 (FGF21) is a promising drug candidate to combat metabolic diseases. However, high-level expression and purification of recombinant FGF21 (rFGF21) in *Escherichia coli (E. coli) *is difficult because rFGF21 forms inclusion bodies in the bacteria making it difficult to purify and obtain high concentrations of bioactive rFGF21. To overcome this problem, we fused the *FGF21 *with *SUMO *(Small ubiquitin-related modifier) by polymerase chain reaction (PCR), and expressed the fused gene in *E. coli *BL21(DE3).

**Results:**

By inducing with IPTG, SUMO-FGF21 was expressed at a high level. Its concentration reached 30% of total protein, and exceeded 95% of all soluble proteins. The fused protein was purified by DEAE sepharose FF and Ni-NTA affinity chromatography. Once cleaved by the SUMO protease, the purity of rFGF21 by high performance liquid chromatography (HPLC) was shown to be higher than 96% with low endotoxin level (<1.0 EU/ml). The results of *in vivo *animal experiments showed that rFGF21 produced by using this method, could decrease the concentration of plasma glucose in diabetic rats by streptozotocin (STZ) injection.

**Conclusions:**

This study demonstrated that SUMO, when fused with FGF21, was able to promote its soluble expression of the latter in *E. coli*, making it more convenient to purify rFGF21 than previously. This may be a better method to produce rFGF21 for pharmaceutical research and development.

## Background

FGF21 is a potent regulator of glucose homeostasis [1]. It was originally identified as a hormone that stimulates glucose uptake in adipocytes [2]. FGF21 is induced and secreted from the liver upon fasting and acts on adipose tissues to induce metabolic adaptation to fasting[3,4]. Specifically, FGF21 stimulates lipolysis in adipocytes, a process which releases fatty acids into the bloodstream; when they reach the liver, these fatty acids are converted to ketones[3]. FGF21 is free of the proliferative and tumorigenic effects [5-7] that are documented for other members of the FGF family [3,8,9]. Systemic administration of FGF21 reduced plasma glucose, fructosamine, triglycerides, insulin, and glucagon in diabetic rhesus monkeys[10]. FGF21 administration led to significant improvements in lipoprotein profiles, by lowering low-density cholesterol and by raising high-density cholesterol, as well as causing weight loss in the animals [10].

In recent years, FGF21 has been described as potential new drug candidate to combat metabolic diseases [5,6]. However, producing FGF21 by traditional techniques, such as plasmid recombination in *E. coli*, yields disappointing results. Kharitonenkov's experiment has shown that recombinant FGF21 (rFGF21) accumulated and formed inclusion bodies in transformed *E. coli *[5]. Our previous experiments also showed that rFGF21 without fusion expression more easily formed inclusion bodies, and it was difficult to produce bioactive protein (data not shown), because the inclusion bodies need to be denatured, annealed, and purified through many chromatography columns.

SUMO (Small ubiquitin-related modifier) proteins are covalently attached to and removed from specific protein substrates in eukaryotic cells[11]. Sumoylation as a reversible post-translational modification process has been shown to be involved in many cellular processes including nuclear-cytosolic transport, apoptosis, protein activation, protein stability, stress response, and cell cycle progression[11,12]. In recent years, SUMO has become an effective biotechnological tool as a fusion system to enhance soluble expression of proteins and decrease proteolytic degradation, which could not be achieved by traditional expression systems[13,14]. SUMO is then post-translationally enzymatically cleaved from the desired protein by SUMO C-terminal hydrolases-isopeptidases [13]. Proteins such as, SARS virus protein[15], MMP13[16], EGF[17], metallothionein[18], and KGF2[19] have been successfully expressed and purified using this fusion strategy.

Recently, Ren [20] expressed FGF21 using a commercial vector containing a SUMO tag and showed that SUMO promotes the soluble expression of FGF21 in *E. coli*. However, no higher level of purity was attained using Ni-NTA affility purification alone. Thus, we cloned SUMO fragment and constructed an expression plasmid containing SUMO and human FGF21. The results show that this novel method of protein expression can promote significantly higher rFGF21 levels, making it easier to be dissolved in the soluble fraction for purification and producing native N-terminal recombinant protein with its bioactivity preserved.

## Results and discussion

### Synthesis of the fusion gene and construction of pET-SUMO-FGF21 expression vector

**Figure 1:**
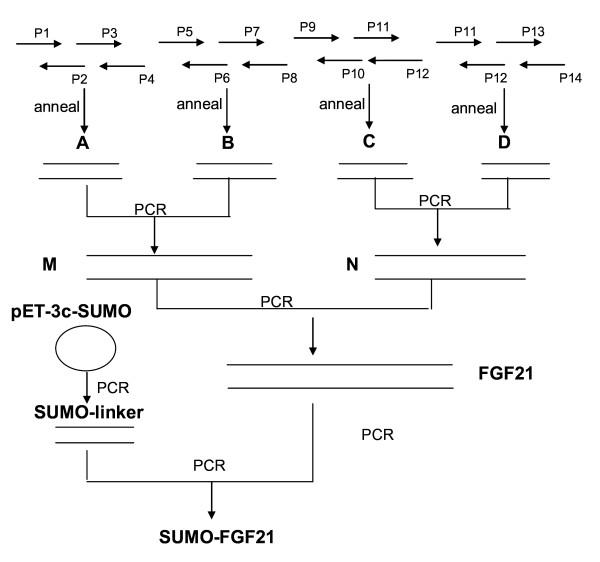
**Schematic illustration of SUMO-FGF21 synthesis**. According to the human FGF21 mRNA coding sequence and the SUMO fragment C-terminal sequence, 21 primers were designed and synthesized. As shown in Figure 1, the SUMO-FGF21 was synthesized by primer extension and PCR. The detailed steps are reported in *Material and Methods*.

**Figure 2:**
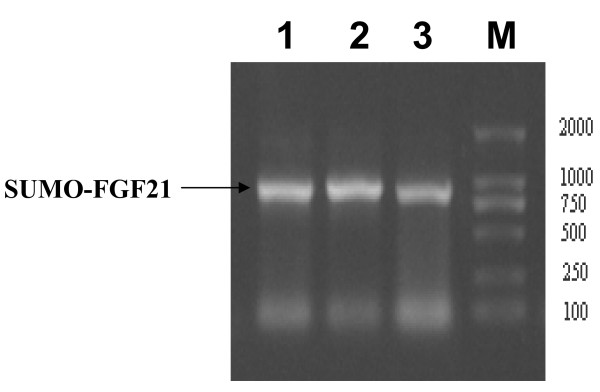
**Synthesis of SUMO-FGF21 by PCR**. The strategy for synthesizing the fusion fragment is described in *Materials and Methods*. The molecular weight of the PCR fragment containing SUMO-FGF21 is shown in Lane 1, 2 and 3. It is 864 bp in length.

To synthesize the fusion gene composed of SUMO and FGF21, fourteen special primers were designed (Table 1). The synthesis strategy is described in the Figure 1 and the detailed procedure is included in *Materials and Methods*. The PCR products of SUMO-FGF21 are shown in Figure 2. The final PCR product was digested with two restriction enzymes (*Nde*I and *Xho*I) and cloned into the expression vector pET-20b(+). The sequence of the recombinant plasmid was confirmed by automated DNA sequencing. The results show that the sequence of the SUMO-FGF21 (864 bp, SUMO 318 bp, human FGF21 546 bp) is in conformity with the prior sequence. The sequence of the SUMO-FGF21 has been submitted the GenBank database (accession number: GU456634).

**Table 1:** PCR primers for amplifying the SUMO-FGF21 fusion gene

Primer name (size)	Sequence (5'- 3)
Pa(32 bp)	ggaattccatatgcatcatcatcatcatcacg
Sumo linker(39 bp)	tggagtcagggatggggtgaccaccaatctgttctctgt
P1(59 bp)	gccggatccatgcaccccatccctgactccagtcctctcctgcaattcgggggccaagt
P2(59 bp)	ctgtctgctgggcatcatctgtgtagaggtaccgctgccggacttgcccccg aattgc
P3(59 bp)	gatgatgcccagcagacagaagcccacctggagatcagggaggatgggacggtgggggg
P4(59 bp)	tttcagctgcaggagactttcggggctctggtcagcagcgccccccaccgtcccatcct
P5(59 bp)	aagtctcctgcagctgaaagccttgaagccgggagttattcaaatcttgggagtcaaga
P6(59 bp)	tacagggccccatctggccgctggcacaggaacctggatgtcttgactcccaagatttg
P7(59 bp)	ggccagatggggccctgtatggatcgctccactttgaccctgaggcctgcagcttccgg
P8(59 bp)	cggactggtaaacattgtatccgtcctcaagaagcagctcccggaagctgcaggcctca
P9(59 bp)	tacaatgttaccagtccgaagcccacggcctcccgctgcacctgccagggaacaagtc
P10(59 bp)	gaagcgagctggtcctcggggtgcagggtcccggtgtggggacttgttccctggcaggt
P11(59 bp)	ccgaggaccagctcgcttcctgccactaccaggcctgccccccgcactcccggagccac
P12(59 bp)	gaggagcccacatcggggggctggggggccaggattccgggtggctccgggagtgcggg
P13(59 bp)	cccccgatgtgggctcctcggaccctctgagcatggtgggaccttcccagggccgaagc
P14(46 bp)	ccgctcgagtcaggaagcgtagctggggcttcggccctgggaaggt

### Expression and purification of SUMO-FGF21

**Figure 3:**
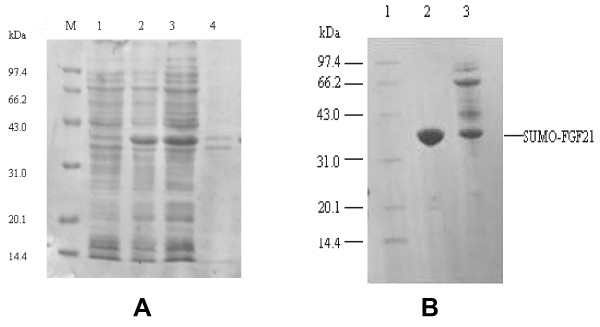
**SDS-PAGE analysis of expression and purification of SUMO-FGF21**. Bacterial containing pET-SUMO-FGF21 were induced by IPTG for 3 h or 4 h at 37°C in Figure 3A. The results from the different treatments are shown here. M: protein molecular weight markers;*Lane 1*: the supernatant of BL21 (DE3) containing pET-SUMO-FGF21 without IPTG;*Lane 2*: the supernatant of BL21 (DE3) containing pET- SUMO-FGF21 induced with 1 mM IPTG for 3 h; *Lane 3*: the supernatant of BL21 (DE3) containing pET- SUMO-FGF21 induced with 1 mM IPTG for 4 h; *Lane 4*: the precipitation of BL21 (DE3) containing pET-SUMO-FGF21 induced with 1 mM IPTG for 4 h. After recombinant bacteria were treated by sonication and centrifugation, the supernatants were loaded on a DEAE sepharose FF and Ni-NTA orderly. The purification results for SUMO-FGF21 are shown in the Figure 3B. *Lane1*: protein molecular weight marker, *Lane2*: purified SUMO-FGF21 eluted from Ni-NTA column; *Lane3*: SUMO-FGF21 eluted from DEAE sepharose FF column.

Recombinants were inoculated in fresh Luria-Bertani (LB) medium, and incubated in a shaking incubator at 37°C until the OD_600 _was 1.0 to 1.2. Isopropyl -D-thiogalactoside (IPTG) was then added to a final concentration of 1 mM for the induction of expression. Recombinant bacterial cells were collected by centrifugation and lysed by sonication. The supernatants and pellets were collected and subjected to 12% SDS-polyacrylamide gel electrophoresis (SDS-PAGE) analysis. The results show that the molecular weight of the expression product is 37 kDa, which corresponds to the predicted size of SUMO-FGF21. The target protein is more than 30% of total cellular protein and the soluble fraction reaches 95.2% of the total expressed recombinant proteins (Figure 3A).

According to the isoelectric point of fusion protein, DEAE Sepharose FF was chosen for the purification of SUMO-FGF21. SDS-PAGE results show that most of the host proteins are removed from SUMO-FGF21 after being purified with a DEAE Sepharose FF column. Furthermore, a Ni-NTA affinity column was chosen for further purification. Proteins without 6 × His tags were removed from the Ni-NTA resin using PBS containing 10 mM and 20 mM imidazole; SUMO-FGF21 was eluted using PBS containing 300 mM imidazole. SDS-PAGE analysis of samples taken from this step showed that the purity of SUMO-FGF21 reached 96.5% (Figure 3B).

In recent years, there have been many reports concerning SUMO as an expression fusion tag. The expression level of the fusion protein containing metallothionein, GST and SUMO was 38.4% of the total supernatant proteins from the organism [18]. Our data show that SUMO-FGF21 concentration is over 25% of all soluble proteins. Expression efficiency may be affected by molecular weight, amino acid constitution, and the function of proteins, etc. As far as protein isolation, we performed two-step purifications, which resulted in the purity of SUMO-FGF21 reaching 96.5%. The results show that two-step purification is helpful for improving the purity of rFGF21, which was more successful than one step Ni-NTA purification [20], as well as inclusion body purification [21].

### Cleavage of the SUMO domain from SUMO-FGF21 protein and further purification of rFGF21

**Figure 4:**
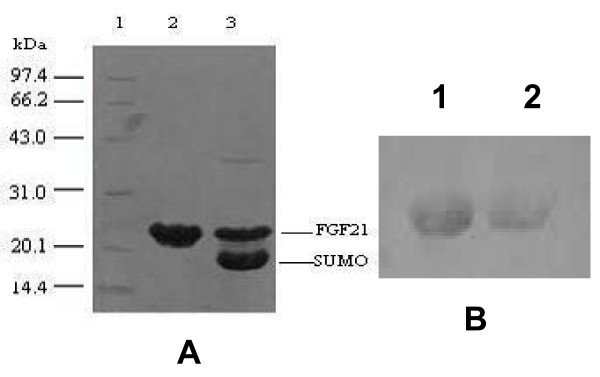
**SDS-PAGE analysis of SUMO-FGF21 by SUMO protease and rFGF21 assay by Western blot**. The purified SUMO-FGF21 was digested by SUMO protease at 4°C overnight. As seen in Figure 4A, rFGF21 was cut from the SUMO-FGF21 by SUMO protease. *Lane1*: Protein Molecular Weight Marker. *Lane2*: purified rFGF21;*Lane3*: SUMO-FGF21 fusion protein after treatment with SUMO protease. Western blot analysis was performed as described in *Materials and Methods*. Immunoacitivity of purified rFGF21 with anti-human FGF21 antibody is shown in Figure 4B. *Lane1*: standard FGF21 (PeproTech Comp.);*Lane2*: purified rFGF21.

**Figure 5:**
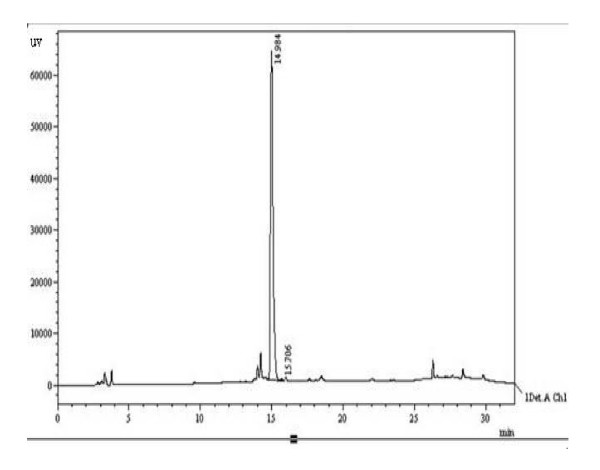
**HPLC analysis of purified rFGF21**. The purity of rFGF21 eluted from a Ni-NTA sepharose column was further evaluated by HPLC analysis using a C18 column. As seen from the chromatogram, the *y*-axis indicates the absorbance, while the *x*-axis represents elution time (min). The main peak observed at 14.984 min corresponds to rFGF21.

When the target protein was fused directly to the C-terminus of SUMO, cleavage by SUMO protease 1 resulted in the release of the target protein with the desired N-terminal amino acid sequence[16,22]. In our studies, the pre-purified fusion protein was diluted and cleaved by SUMO protease. The lysate was purified by Ni-NTA resin. SUMO and SUMO protease containing His tags that were affiliated by Ni-NTA resin, but rFGF21 was eluted out of the Ni-NTA column. The results showed that rFGF21 was highly purified by SDS-PAGE (Figure 4A) and could react with the human FGF21 polyclonal antibody by Western blot (Figure 4B). HPLC analysis of the target protein showed a major peak of rFGF21, with a retention time of 14.984 min; the purity exceeded 96% (Figure 5).

Next, we tested endotoxin levels from three different batches of purified rFGF21, and the results were less than 1.0 EU/ml, which is considered acceptable compared to commercially available proteins. These levels were also in accordance with the standard of injection biologics listed in the Chinese Pharmacopeia of 2005. It also suggests that the purity of rFGF21 by this method can meet the requirements of pharmaceutical research *in vivo*.

### Mass spectrometric and N-terminal amino acid sequence analysis

In order to confirm the authenticity of rFGF21, the target protein was subjected to MALDI-TOF mass spectroscopy analysis and N-terminal amino acid sequencing analysis. The molecular weight of rFGF21 analyzed by MALDI-TOF mass spectroscopy was 19.424 kDa, which was very close to 19.411 kDa, theoretical molecular weight of FGF21. Moreover, according to the sequencing result, the first five amino acids of our rFGF21 N-terminus were H-P-I-P-D, which was in conformity with native FGF21.

### *In vivo *bioactivity of rFGF21

**Figure 6:**
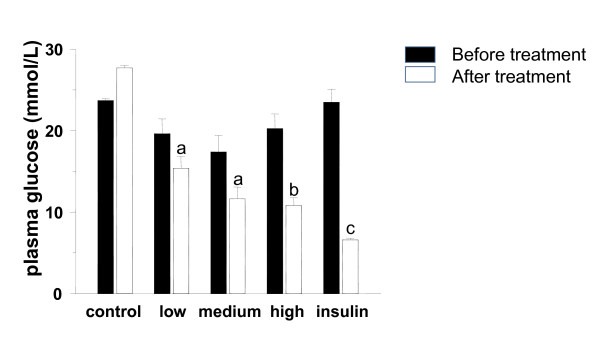
**The effects of rFGF21 on reducing plasma glucose in diabetic rats**. The STZ treated diabetic rats were injected with different doses of rFGF21 once daily for two weeks. Control: 0.9% NaCl; Low: 0.15 mg/kg rFGF21; Medium: 0.3 mg/kg rFGF21; High: 0.6 mg/kg rFGF21; Insulin: 5U per rat. The results show that rFGF21 acts in a dose-dependent manner to reduce plasma glucose in diabetic rats, with the high dose rFGF21 demonstrating better effects than the other doses. Compared to rFGF21, insulin displays a more significant effect in decreasing plasma glucose. ^*a*^*P *< 0.05, ^*b*^*P *< 0.01 and ^*c*^*P *< 0.001 were considered as the significant difference for post-treatment compared to pre-treatment.

The construction of diabetic rat models is described in *Materials and Methods*. We tested the effects of rFGF21 on diabetic rats. Diabetic rats were treated daily for two weeks with high, medium, and low dosages of rFGF21 (150, 300, 600 μg/kg/d). All treatments produced significant changes when compared with the control animals. The statistical results are described using column graph (Figure 6). We can see that the changes in plasma glucose levels all show significant differences compared to controls after injecting three doses of rFGF21(^*low*^*P *< 0.05 vs control, ^*medium*^*P *< 0.05 vs control, ^*high*^*P *< 0.01 vs control). The results also show that rFGF21 acts in a dose-dependent manner to reduce plasma glucose in diabetic rats. High doses of rFGF21 result in better effects than the other two doses. The high dose lowers circulating glucose from 20.25 ± 5.13 mmol/L to 10.83 ± 2.73 mmol/L. Compared to rFGF21, insulin displayed more significant effects in decreasing plasma glucose with ^*c*^*P *< 0.001, which is considered a significant difference between pre- and post-treatment. The different effects might be due to different mechanism in decreasing plasma glucose between insulin and FGF21. In contrast to insulin, which is known to function via GLUT4 translocation, FGF-21 primarily act through up-regulation of cellular GLUT1[23].

Many other experiments have also documented that FGF21 could promote the glucose uptake in adipocytes [6] and 3T3-L1 cells [5], as well as reduce plasma glucose and triglyceride in animal models of type 2 diabetes, such as mouse and monkey [20,24]. However, there were some differences in rFGF21-mediated reductions in blood glucose levels attributable to different animal genotypes and treatment. In *ob/ob *mice, doses of 125 and 750 μg/kg/d of FGF-21 significantly lowered blood glucose compared with vehicle treatment after 3 days of administration[5]. After 8 days of drug administration, FGF21 treatment not only reduces fasting blood glucose, but also improves insulin sensitivity in *ob*/*ob *mice [24]. Notably, 100 μg/kg dose of rFGF-21 lowered mean fasting plasma glucose from the overtly diabetic level to near normal when administered for 6 weeks [10]. In our studies, rFGF21 at doses of 150, 300 or 600 μg/kg/d all decreased glucose levels in diabetic mice. Administration of FGF21 resulted in a dose-dependent reduction in circulating levels of glucose.

## Conclusions

This study demonstrates that, when fused with SUMO, soluble expression of rFGF21 increased in *E. coli*; this form is also conveniently isolated rFGF21 than previously described forms. As a novel molecular chaperon domain, SUMO increases the yield of recombinant protein and makes it easier to dissolve in the soluble fraction for protein purification. This strategy can be used as a source of reference for the expression and purification of other proteins.

## Methods

### Reagents

*Pyrobest*^® ^DNA Polymerase and restriction enzymes *Nde*I and *Xho*I were purchased from TaKaRa Company (Japan). PCR purification kit, gel extraction kit, and plasmid miniprep kit were obtained from the Omega Company (USA). The expression vector pET-3c-SUMO was produced by Engineering Research Center of Bioreactor and Pharmaceutical Development, Ministry of Education, Jilin Agricultural University (China). DEAE Sepharose FF column and Sepharose G50 column were purchased from GE healthcare (Sweden). Ni-NTA agarose was obtained from Invitrogen (USA). Human FGF21 protein was purchased from the PeproTech Company (Korea), and anti-hFGF21 antibody was purchased from Santa Cruz Biotechnology Company (USA); Dr. Yadong Huang from Ji Nan University graciously supplied us with the SUMO protease.

### Construction of pET- SUMO-FGF21 recombinant plasmid

The strategy for synthesizing the fusion gene is illustrated in Figure 1. The core fragment of SUMO was first obtained by polymerase chain reaction (PCR), which was performed as follows: reaction mixture containing P1, P2, P3, P4, dNTP and 10 × PCR buffer were incubated at 94°C for 4 min. *Pyrobest*^® ^DNA polymerase was added to the reaction when the temperature decreased to 55°C. The annealing step was conducted at 55°C for 5 min and elongation was done at 72°C for 60 sec to get fragment A. Likewise, P5, P6, P7, and P8, were reacted to form fragment B; primers P9, P10, P11, and P12 formed fragment C, primers P11, P12, P13, and P14 formed fragment D. Using A and B as the common template, and P1 and P8 as the forward and reverse primers, the reaction was denatured at 94°C for 5 min. This was followed by 30 consecutive cycles of denaturation: 95°C for 30 s, annealing for 30 s at 55°C, extension at 72°C for 1 min, and then a final single extension at 72°C for 7 min to get the fragment M. Fragments C and D were used to create fragment N. Finally, Fragments M and N were used to form the FGF21 fragment, according to the previously described procedure.

Using pET3c-SUMO (plasmid containing SUMO fragment) as template, Pa and the SUMO linker as the forward and reverse primers, the SUMO-linker was generated. Then, using the SUMO-linker and the FGF21 fragment as the common template, Pa and P14 as the forward and reverse primers, the SUMO-FGF21 full-length constructed was created using the same PCR reaction procedure described above.

The fusion gene was digested with *Nde*I and *Xho*I, and then ligated into previously digested pET-20b(+) to create the new expression vector, pET-SUMO-FGF21. Authenticity of the inserted fragment was confirmed by restriction enzyme analysis and automated DNA sequencing.

### Expression and soluble detection of SUMO-FGF21

The recombinant plasmid, pET-SUMO-FGF21 was transformed into *Escherichia coli *BL21 (DE3) cells. SUMO-FGF21 was expressed as follows: a single transformed colony was grown in 5 ml of LB medium (1% peptone, 1% yeast extract, and 0.5% sodium chloride, pH 7.0) containing 100 μg/ml ampicillin at 37°C with shaking at 250 rpm. After overnight growth, 500 μl of culture was transferred into 50 ml of fresh LB medium for exponential growth. When the OD_600 _value reached 0.6, isopropyl-β-D-thiogalactopyranoside (IPTG) was added to the culture at a final concentration of 1 mmol/L. The culture was then incubated for another 4 hours at 37°C with shaking at 220 rpm. SUMO-FGF21 was separated by 12% sodium dodecyl sulfate polyacrylamide gel electrophoresis (SDS-PAGE), and the amount of fusion protein yield was measured by densitometer scanning.

Cell medium induced under different conditions was harvested by centrifugation at 8000 rpm for 5 min. Cell pellets were suspended into 20 mmol/L PBS buffer and dissolved by sonication. The suspensions were centrifuged at 18,000 rpm for 30 min at 4°C. The clear supernatant was collected (soluble fraction) and the remaining pellets (insoluble fraction) containing inclusion bodies were resuspended into an equal volume of lysis buffer. Both soluble and insoluble fractions were analyzed by 12% SDS-PAGE.

### Purification of SUMO-FGF21

The cell lysate was purified by DEAE-Sepharose FF column chromatography. SUMO-FGF21 was eluated with buffer containing different concentrations of NaCl (100-300 mmol/L, pH 9.0), followed by further purification with Ni-NTA resin (Invitrogen). The Ni-NTA resin was washed with wash buffer I (20 mmol/L PBS containing 10 mM imidazole and 150 mM NaCl, pH 8.0) until OD_280 _of effluent reached base line. Contaminating proteins were eluted from the column with wash buffer II (20 mmol/L PBS containing 20 mM imidazole and 150 mM NaCl, pH 8.0). Finally, 6 × His-tagged SUMO-FGF21 protein was collected from the column with elution buffer (20 mmol/L PBS containing 300 mM imidazole and 150 mM NaCl, pH 8.0). Samples taken at the elution peak were pooled. The purity of SUMO-FGF21 was assessed using SDS-PAGE and the concentration was evaluated by the Bradford method.

### Cleavage of SUMO from SUMO-FGF21 and purification of rFGF21

The eluted fusion protein was diluted to a concentration of 1 mg/ml. Ten unit of SUMO protease were added to the dilution and the mixture was incubated in high salt buffer (50 mM Tris-HCl, pH 8.0, 0.15 M NaCl, 1 mM DTT) for 1 hr at 4°C. Following incubation, the mixture was loaded onto an Ni-NTA resin column to obtain the rFGF21, which was further concentrated by ultrafiltration at 4°C. The SUMO, SUMO-FGF21 and SUMO protease stayed bound to the Ni-NTA resin and only rFGF21 was eluted from the column when washed off the resin with elution buffer. rFGF21 was then desalted with Sepharose G50 column.

### The authenticity assay for rFGF21

The immunogenic activity of purified rFGF21 was assayed by Western blotting. Total cellular protein was boiled in an equal volume of sample loading buffer, a Tris-HCl buffer (pH 6.8) containing 20% glycerol, 2.5% SDS, 10% β-Mercaptoethanol and 0.005% bromophenol blue. Protein samples were electrophoresed on 12% polyacrylamide gels containing SDS. Proteins were electrophoretically transferred onto PVDF membranes, and after overnight exposure to 3% BSA to block nonspecific binding, the membranes were incubated with a polyclonal FGF21 antibody (Santa Cruz Biotechnology Inc, 1:1000). The membranes were washed and incubated with a 1:5000 dilution of secondary HRP-conjugated antibody (rabbit anti-goat IgG, Amersham Biosciences Corp.). Immunoreactive bands were visualized using a DAB kit (Boshide Inc, Wuhan, China).

For high performance liquid chromatography (HPLC) analysis, purified rFGF21 was desalted and then loaded onto a C18 column. The elution was carried out using a linear gradient of 30-70% acetonitrile at a flow rate of 0.8 ml/min in the presence of 0.1% trifluoroacetic acid (TFA). The fractions containing rFGF21 were pooled and subjected to N-terminal amino acid sequencing, amino acid composition analysis and MALDI-TOF mass spectroscopy analysis. The concentration of rFGF21 was evaluated by the Bradford method.

### Endotoxin assay

The endotoxin concentration of rFGF21 was estimated quantitatively using the Chromogenic Tachypleus Amebocyte Lysate (TAL) endpoint assay kit as described by the manufacturer (Zhanjiang A&C Biological Ltd, China). The assay was performed according to the kit protocol.

### *In vivo *activity assay of rFGF21

All procedures involving animals and their care described here were approved by the Institutional Animal Care and Use Committee of Jilin Agricultural University and were performed in accordance with institutional guidelines for animal experiments. Male Wistar adult rats (weighting at 180-220 g) were randomly divided into the high fat diet group (HFD, *n *= 40) and the common diet group (NC, *n *= 8) after being fed a high fat diet for two months. HFD group animals were given peritoneal injections of 35 mg/kg streptozotocin (STZ, Sigma, USA), while the NC group was given an equivalent volume of citric acid buffer. One week later, blood was collected and the fasting plasma glucose (FPG) was assayed. STZ treated rats with FPG = 11.1 mmol/L were chosen as type 2 diabetes models (*n *= 40). Subsequently, 32 diabetic rats were divided into five groups (n = 8/group): 1) the diabetic rats treated with a 0.6 mg/kg of rFGF21 group (high dose); 2) diabetic rats treated with a 0.3 mg/kg of rFGF21 group (medium dose); 3) diabetic rats treated with a 0.15 mg/kg of rFGF21 group (low dose); 4) diabetic rats treated with insulin (5 U/day) group (positive control) and 5) diabetic rats treated with 0.9% NaCl group (negative control). In addition, the 8 NC rats constituted the blank control group. Drugs were given by subcutaneous injection once daily for two weeks.

### Statistical analysis

Values are expressed as means ± S.E.M. Mean Value Comparisons between two groups were performed using the *Student t test*. Significant differences were considered at *P *< 0.05.

## Competing interests

The authors declare that they have no competing interests.

## Authors' contributions

HYW performed the main experiments of this project, including rFGF21 purification, immunoblot assay and activity analysis. YCX conceived the idea, directed the graduate student and drafted the manuscript. LJF and HXZ participated in the purification of rFGF21. YFZ participated in the cloning of the fusion gene and constructing the expression plasmid. XSW and HCG performed *in vivo *activity assay of rFGF21. YDH supported the SUMO protease and revised the manuscript. XKL, as the Principal Investigator of the project, revised the manuscript. All authors have read and approved the manuscript.
